# A mass conundrum

**DOI:** 10.1093/jscr/rjad500

**Published:** 2023-09-05

**Authors:** Tan Jun Guang Kendric, Sabu Thomas

**Affiliations:** General Surgery, Kalgoorlie Health Campus, 15 Piccadilly St, Kalgoorlie 6430, Australia; General Surgery, Kalgoorlie Health Campus, 15 Piccadilly St, Kalgoorlie 6430, Australia

**Keywords:** omental torsion, bowel obstruction, colorectal

## Abstract

A 30-year-old female presented to a rural hospital in Western Australia complaining of generalized abdominal pain and symptoms of partial bowel obstruction. Computed tomography (CT) showed a radiological ‘whirl’ sign, with images 4 years apart demonstrating interval progression. Given our patient’s past history with miliary tuberculosis, we performed a diagnostic laparotomy which confirmed omental torsion. We report a case of surgically and pathologically proven chronic secondary omental torsion presenting with partial bowel obstruction, and showcase rarely seen interval progression on CT.

## Introduction

Omental torsion was first described in 1866 by Eitel [1]. To date, it is still a relatively rare condition and given the non-specific nature of presentation, is commonly diagnosed intra-operatively. Omental torsion is categorized into either primary or secondary, with the latter being more common. Primary omental torsion is unipolar, with one end fixed and the opposing end free. Etiology is generally unclear. Predisposing factors include anatomical variations where primary omental torsion more commonly occurs at the right side, reflecting the omentum’s larger size on the right. Obesity can cause irregular fatty omental deposits, predisposing to primary torsion. Events that incite a torsion event have been hypothesized to be bowel hyperperistalsis and blunt trauma. Secondary omental torsion is usually bipolar, which is torsion of a central portion between two fixed points. This is commonly from adhesions, joining the free omental end to the peritoneum or other intra-abdominal structures [2, 3]. Once torsion occurs, it is usually complete and permanent. Venous return becomes restricted and distal omentum becomes congested. A characteristic serosanguineous fluid in the peritoneal cavity is a result of hemorrhagic extravasation. If torsion continues, hemorrhagic infarction and necrosis eventually occurs [4]. Symptoms are non-specific and can range mimic appendicitis, Meckel’s diverticulitis, right sided diverticulitis, cholecystitis, ovarian torsion, and ectopic pregnancy [5]. Computed tomography (CT) is the main diagnostic tool with a characteristic ‘whirl sign’. Ultrasound may be a useful adjunct to exclude other pathologies. Treatment consist of surgical ligation and resection, which is usually followed by rapid and complete recovery.

## Case report

Our patient is a 30-year-old female who presented to a rural hospital in Western Australia with a 2-month history of recurring abdominal pain, nausea, and vomiting. On further history, she describes a clinical picture of intermittent bowel obstruction, going for days without flatus before self-resolution. This was associated with unintentional 4-kg weight loss and anorexia. On examination, she was vitally stable. She had a lower midline laparotomy scar with no abdominal distension and no palpable masses. Her abdomen was subjectively tender at the right upper and lower quadrants with no peritonism.

Her past medical history was significant for miliary tuberculosis. At age of 19 years, she underwent a diagnostic lower midline laparotomy for drainage of pelvis abscess. Intra-operative findings revealed purulent peritonitis and abdominal tuberculosis. She subsequently completed anti-tubercular treatment in 2010. These were managed in Thimphu, Bhutan where our patient was originally from before her migration to Australia. She takes no regular medications and has no known drug allergies.

On presentation, her blood work showed a neutrophilia of 8.38 (2–7.5) but was otherwise unremarkable with white cell count 10.7 (4–11), CRP 2.6 (<5) and lymphocyte 1.53 (1.2–4).

**Figure 1:**
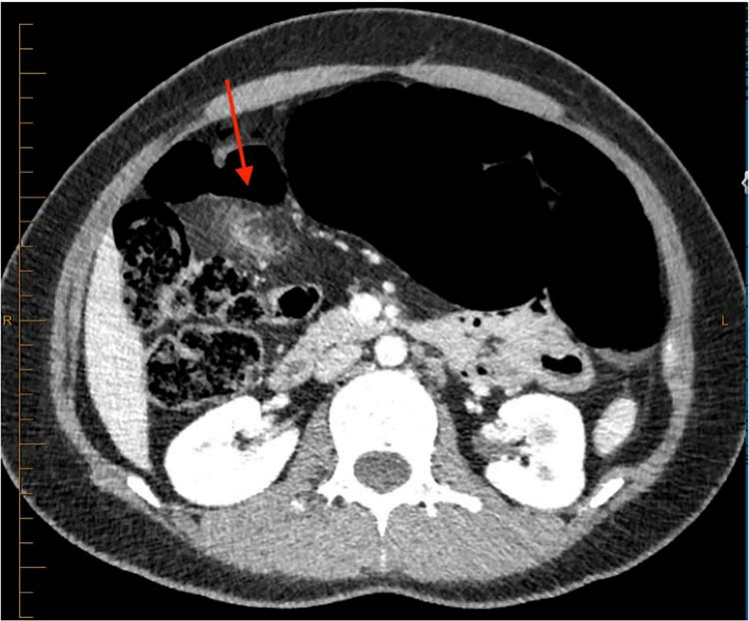
CT abdomen pelvis in 2019 (axial).

**Figure 2:**
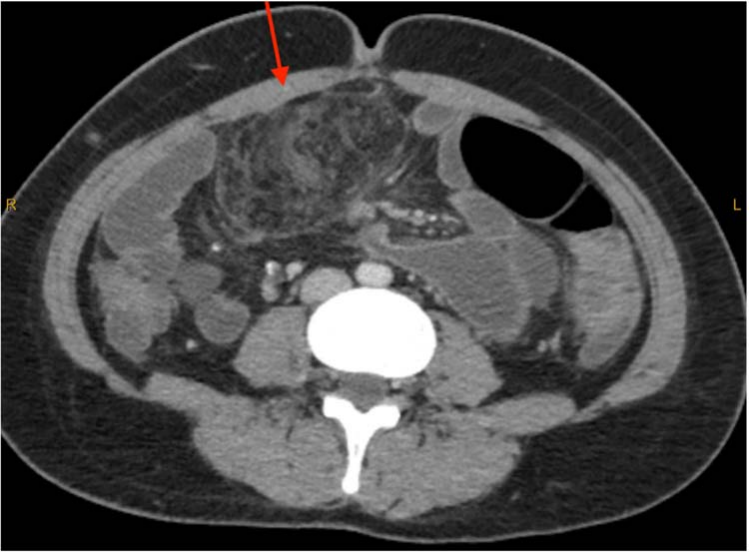
CT abdomen pelvis in 2023 (axial).

**Figure 3:**
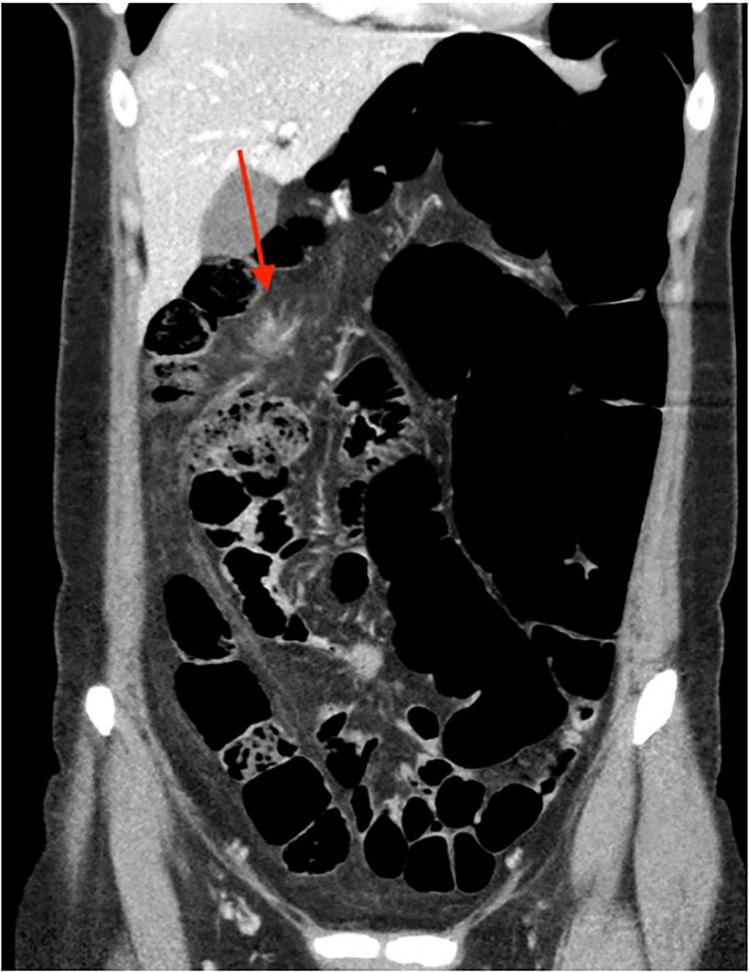
CT abdomen pelvis in 2019 (coronal).

**Figure 4:**
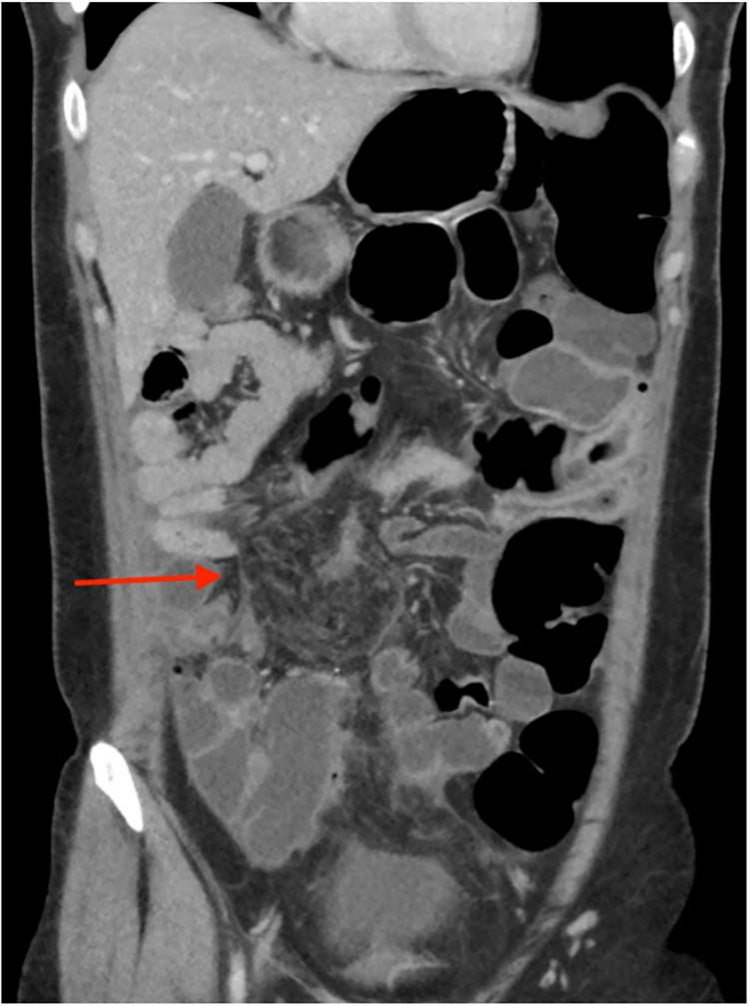
CT abdomen pelvis in 2023 (coronal).

On review of imaging, we noted a CT scan from 2019 which showed swirling and stranding of omentum/mesenteric fat inferior to hepatic flexure (CC 1.8 cm, TV 2.6 cm), with suspicion of internal herniation of the omental/mesenteric fat leading to infarction (Figs 1 and 3). Management of this was unclear as this was at a private hospital in metropolitan Western Australia. A repeat CT scan during her current presentation revealed similar swirling of omentum/mesenteric fat, but with interval increase in size (CC 5.5 cm, TV 7.6 cm). There was associated small bowel dilatation and wall thickening and enhancement with no focal transition point (Figs 2 and 4).

**Figure 5:**
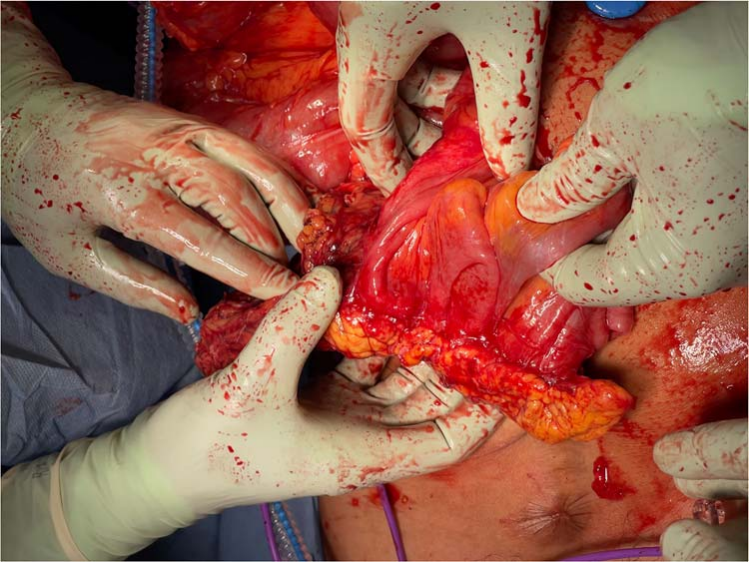
Omental mass with extensive adhesions to small bowel.

**Figure 6:**
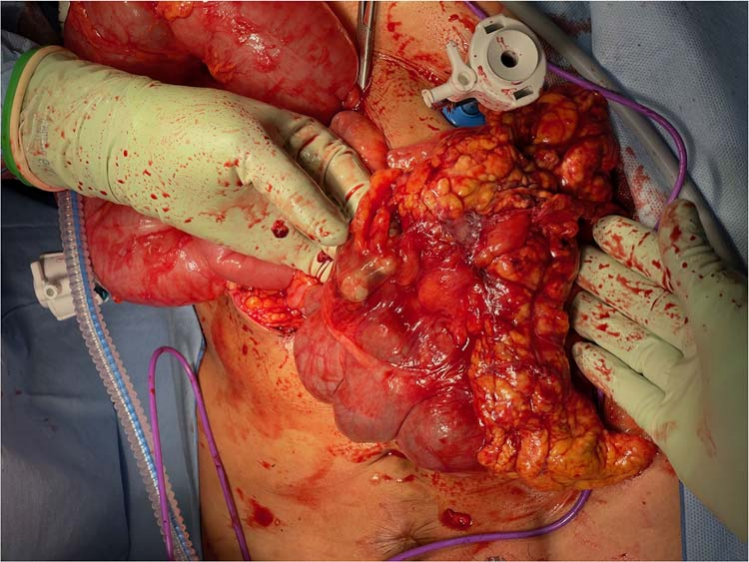
Omental mass with extensive adhesions to ascending colon.

**Figure 7:**
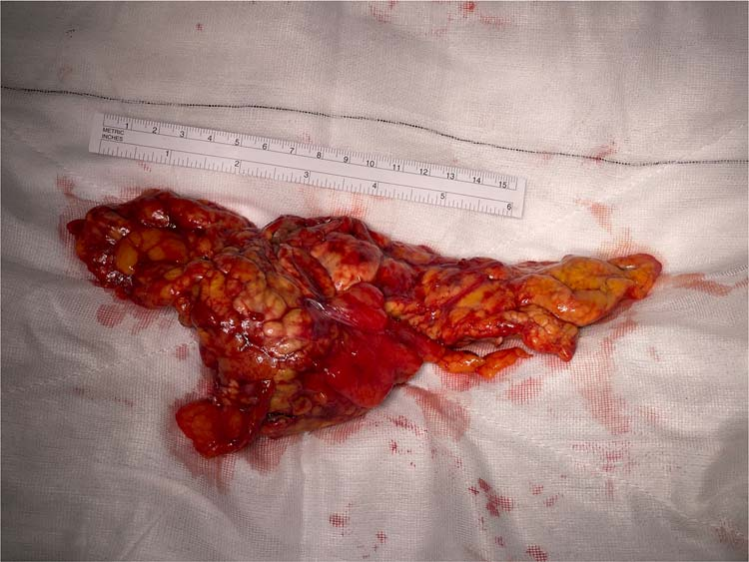
Omental mass.

Given the clinical findings of recurring abdominal pain, imaging suggesting small bowel obstruction with enteritis and the past history of miliary tuberculosis, the decision to proceed with diagnostic laparoscopy was made. On performing Hasson entry, we encountered extensive adhesions which necessitated conversion to laparotomy and prolonged adhesiolysis. We then found a large calcified inflammatory omental mass situated in the mid-abdomen, independent from the surrounding omentum. The mass appeared tethered to the ascending colon, obtaining its blood supply from the mesentry (Fig. 7). It was also tethered with inflammatory adhesions to the peritoneum and surrounding small bowel (Figs 5 and 6), causing significant kinking of the small bowel over a length of about one meter. This was dissected off and the remaining bowel was unremarkable on gross examination.

**Figure 8:**
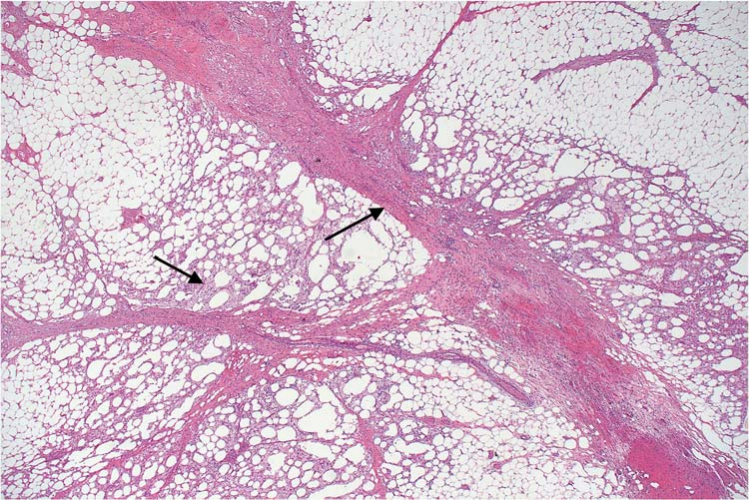
Lower power view of fatty tissue demonstrating a broad band of septal fibrosis, indicating a chronic inflammatory process. Adjacent fat is chronically inflamed and contains a dense infiltrate of foamy histiocytes and some lymphocytes, typical of fat necrosis.

**Figure 9:**
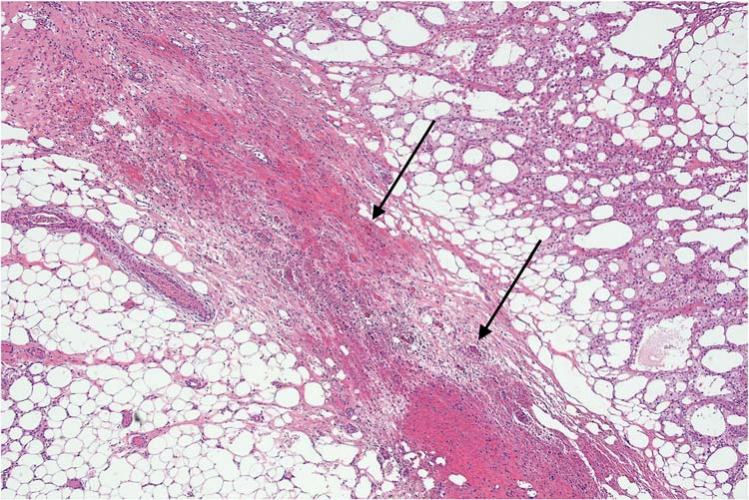
Higher power view demonstrating the thickened fibrous septum with vascular congestion involving small blood vessels.

**Figure 10:**
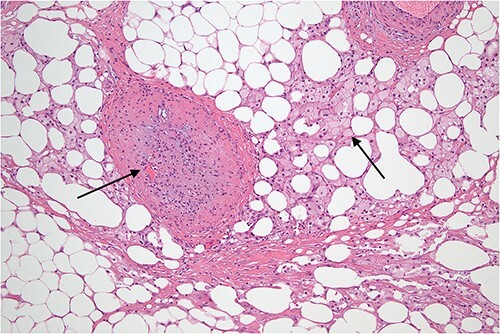
Slightly off-center nodule representing an obstructed medium sized blood vessel with fibrous occlusion of the lumen. There were several small vessels showing similar features in the biopsy indicating a chronic, possibly recurrent process. The necrosis demonstrated in the adjacent fatty tissue is not typical of mycobacterial infection and is what might follow ischemia, possibly related to omental volvulus or herniation and is indicative of a chronic process.

Histopathology revealed omental tissue measuring 170 × 135 × 52 mm with extensive fat necrosis and focal vascular thrombosis causing ischemic necrosis with congestion and some hemorrhage (Figs 8–10), in keeping with torsion and vascular obstruction. The inflammatory changes were not typical of mycobacterial infection and Ziehl–Neelsen staining for acid fast bacilli (mycobacteria) is negative. There was no dysplasia.

At 1-month follow up, our patient reported complete resolution of symptoms.

## Discussion

We believe that our patient may have had chronic secondary omental torsion dating prior to 2019. Her previous abdominal tuberculosis and pelvic abscess may have induced formation of chronic adhesions and triggered a bipolar torsion secondary to adhesions between the free omentum end and intra-abdominal structures (small bowel and ascending colon). Due to the chronicity, the culprit omental mass obtained a secondary blood supply from the large bowel’s mesentry and detached from its original omentum, resulting in an independent omental mass adhering to surrounding bowel loops. The resulting bowel kinks contributed to her symptoms of abdominal pain and recurrent partial obstruction.

Our case is highly unique in multiple aspects. First, our patient presented with atypical signs of secondary omental torsion. In addition to abdominal pain, she reported symptoms in keeping with recurrent partial bowel obstructions. In view of her past miliary tuberculosis, our initial differential diagnosis was an inflammatory phlegmon from potentially a recurrence of intra-abdominal tuberculosis, or encapsulating peritoneal sclerosis. Given her previous exploratory laparotomy, internal herniation from surgically created mesenteric windows was also considered.

Second, omental torsion is rarely managed conservatively with surveillance imaging. Hence, it is rare that we have CT imaging showing the progression of omental torsion over 4 years, noting the markedly increased dimensions and characteristic mesenteric swirling.

In conclusion, omental torsion is a rare cause of abdominal pain that requires a high index of suspicion due to the non-specific presentation. CT scan can serve as a valuable adjunct to decision making, but surgeons should have a low threshold in offering diagnostic laparoscopy for patients failing conservative management.

## Data Availability

No new data were generated or analysed in support of this research.
